# Bacterial translocation and microgap formation at a novel conical indexed implant abutment system for single crowns

**DOI:** 10.1007/s00784-021-04112-2

**Published:** 2021-08-16

**Authors:** Peter Gehrke, Simon Burg, Ulrike Peters, Thomas Beikler, Carsten Fischer, Frank Rupp, Ernst Schweizer, Paul Weigl, Robert Sader, Ralf Smeets, Sogand Schäfer

**Affiliations:** 1 Department of Postgraduate Education, Center for Dentistry and Oral Medicine (Carolinum), University Hospital, Goethe University Frankfurt, 60528 Frankfurt am Main, Germany; 2 Private Practice for Oral Surgery and Implant Dentistry, Bismarckstraße 27, 67059 Ludwigshafen, Germany; 3 Department of Oral and Maxillofacial Surgery, University Medical Center Hamburg-Eppendorf, 20251Hamburg, Germany; 4 Department of Periodontics, Preventive and Restorative Dentistry, University Medical Center Hamburg-Eppendorf, 20251 Hamburg, Germany; 5 Dental Laboratory, Sirius Ceramics, 60528 Frankfurt am Main, Germany; 6 Section Medical Materials Science and Technology, University Hospital Tuebingen, 72076 Tuebingen, Germany; 7 Department for Oral, Cranio-Maxillofacial and Facial Plastic Surgery, Medical Center, University Hospital, Goethe University Frankfurt, 60528 Frankfurt am Main, Germany; 8 Department of Oral and Maxillofacial Surgery, Division of Regenerative Orofacial Medicine, University Hospital Hamburg-Eppendorf, 20251 Hamburg, Germany

**Keywords:** Acuris, Conometric connection, Bacterial leakage, Microgap, Cement gap, Marginal integrity, CAD/CAM crown

## Abstract

**Objectives:**

A conometric concept was recently introduced in which conical implant abutments hold the matching crown copings by friction alone, eliminating the need for cement or screws. The aim of this in vitro study was to assess the presence of microgap formation and bacterial leakage at the Acuris conometric restorative interface of three different implant abutment systems.

**Material and methods:**

A total of 75 Acuris samples of three implant-abutment systems (Ankylos, Astra Tech EV, Xive) were subjected to microbiological (*n* = 60) and scanning electron microscopic (SEM) investigation (*n* = 15). Bacterial migration into and out of the conical coupling system were analyzed in an anaerobic workstation for 48, 96, 144, and 192 h. Bacterial DNA quantification using qrt-PCR was performed at each time point. The precision of the conometric coupling and internal fit of cemented CAD/CAM crowns on corresponding Acuris TiN copings were determined by means of SEM.

**Results:**

qrt-PCR results failed to demonstrate microbial leakage from or into the Acuris system. SEM analysis revealed minute punctate microgaps at the apical aspect of the conometric junction (2.04 to 2.64 µm), while mean cement gaps of 12 to 145 µm were observed at the crown-coping interface.

**Conclusions:**

The prosthetic morse taper connection of all systems examined does not allow bacterial passage. Marginal integrity and internal luting gap between the ceramic crown and the coping remained within the clinically acceptable limits.

**Clinical relevance:**

Conometrically seated single crowns provide sufficient sealing efficiency, relocating potential misfits from the crown-abutment interface to the crown-coping interface.

## Introduction

**Fig. 1:**
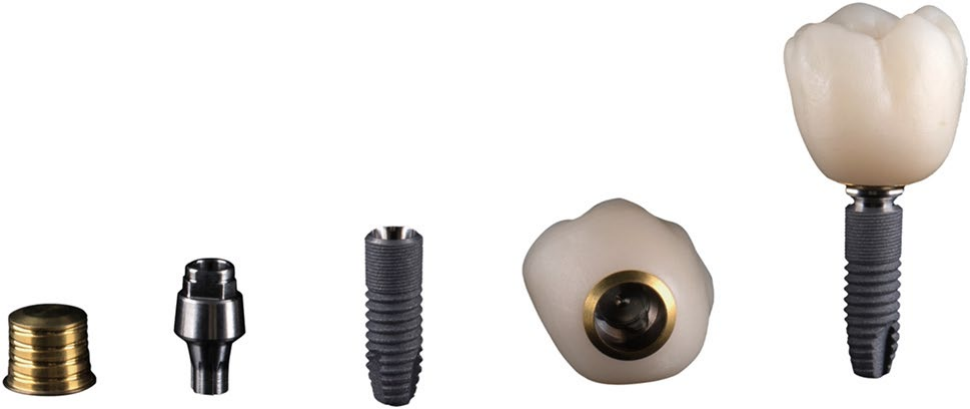
Components of Acuris conical indexed abutment-system illustrated by the example of Astra Tech EV (from left to right): titanium-nitride (TiN) Acuris cap, conical Acuris abutment with anti-rotation connection, implant, extraorally luted all-ceramic crown on TiN cap, conometrically fixed crown-coping complex on assembled abutment

Anchorage of the prosthetic connection for implant-supported fixed dental prostheses (FDPs) is commonly achieved by means of luting cement or screws. To ensure firm retention between multiple implants and the respective superstructure, the use of a conometric concept has been proposed alternatively [1, 2]. In this approach, conical abutments retain matching crown copings solely by surface friction, thus eliminating the need for either cement or screws. Recently, a novel conical indexed abutment (Acuris, Dentsply Sirona Implants, Mölndal, Sweden) with anti-rotation features has been introduced to avoid the undesirable impact of rotational forces in single implant restorations [3, 4]. A modification of this restorative concept from previously published conometric approaches involves extraoral adhesive luting between a titanium nitride-coated (TiN) stock coping (Acuris Cap, Dentsply Sirona Implants, Mölndal, Sweden) and an all-ceramic crown in the dental laboratory, shifting the potential misfits from the crown-abutment interface to the crown-coping interface (Fig. 1). The final crown-coping complex is fixed intraorally to the anti-rotation connection of the conical abutment with an axially directed load of a calibrated striker (Acuris Abutment, Conometric Fixation Tool, both Dentsply Sirona Implants, Mölndal, Sweden). This ensures a correct alignment and secure coupling of the crown. The conometric joint is therefore a fixed retention, with the possibility of maintenance-related disengagement by the dentist.

Unlike traditional fixation methods for the retention of implant-supported reconstructions achieved by either cement- or screw-retaining techniques, the morse taper coupling connection exhibits an inherently superior accuracy, as the FDP abutment connection is achieved by prefabricated, intraorally passivated components. In addition, beneficial clinical outcomes were observed with regard to the prevention of undesirable technical and/or biological prosthetic complications such as screw loosening, fracture, ceramic chipping, debonding of the restoration, undetected cement remnants and subsequent peri-implant tissue inflammation, and/or crestal bone loss [5, 6]. Occlusal openings and related interferences in the veneering surface, as required for a screw-retained approach, are not present.

Despite the promising clinical results for conometric morse taper connections used to retain implant-supported single crowns (SCs) and FPDs, there are limited data on the accuracy of fit at the level of restoration and the potential for bacterial leakage at the conical coupling [7]. A misalignment and resulting microgap between the conometric units could serve as a bacterial reservoir that facilitates plaque formation. This, in turn, may promote inflammation of the peri-implant tissues and crestal bone resorption. Furthermore, the interaction between metal surfaces and the oral environment may lead to the release of implant degradation products into the peri-implant sulcus, which exposes the microbiota to increased environmental stress and subsequently change immune responses to bacteria [8, 9]. A recent pilot study on the microbiological sealing of the novel Acuris junction revealed no bacterial translocation at the conometric interface [10]. However, since this investigation only examined a relatively small number of specimens for a single implant system, verification of these results in a larger group of specimens for different implant abutment configurations is required. The marginal integrity and internal fit of the extraorally luted ceramic crowns on the matching copings is yet to be verified.

The aim of the present in vitro trial was therefore to evaluate the bacterial leak proofing along the conometric junction of 3 different implant-abutment systems for single crown restorations. A secondary objective of the study was to assess the conometric fit as well as the marginal adaption of computer-assisted design and computer-assisted (CAD/CAM) fabricated all-ceramic crowns on the Acuris TiN copings using scanning electron microscopy (SEM). The hypothesis tested was that the cone-in-cone coupling exhibits no detectable microgap and does not allow bacterial translocation, irrespective of the implant-abutment system. Furthermore, it was hypothesized that no difference would be observed between the 3 test groups in terms of internal fit and marginal integrity of the crown-coping interface.

## Materials and methods

### General study setup

**Fig. 2:**
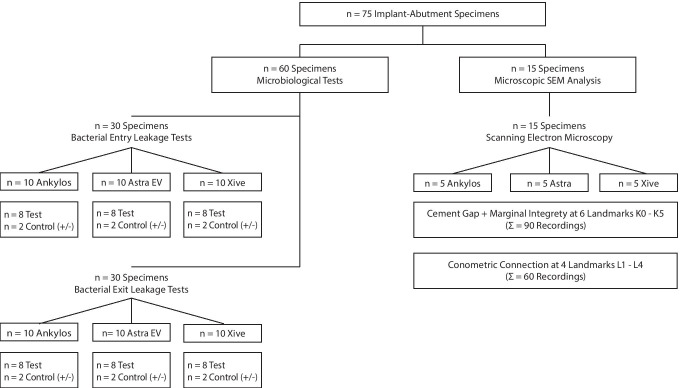
Study design of qrt-PCR microbiological analyses and microscopic examination by means of SEM

**Fig. 3:**
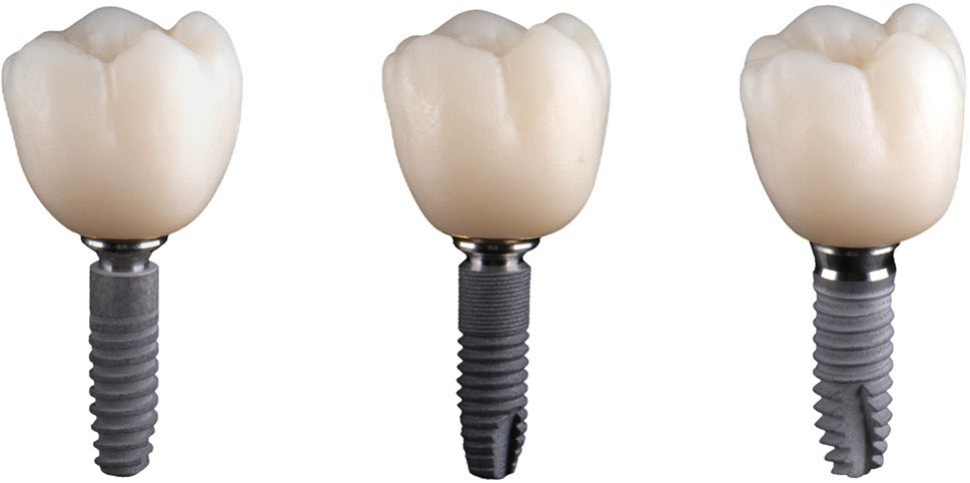
Assembled specimens of the tested implant-abutment systems (from left to right): Ankylos C/X, Astra Tech EV, Xive S plus

A dual study approach was designed to evaluate bacterial leakage along the Acuris morse taper junction and to determine its conometric fit as well as the internal and marginal integrity between the Acuris TiN coping and all-ceramic crown. The principal scheme of the test setup is shown in Fig. 2. A total of 75 conometric samples of three different implant-abutment systems (Ankylos C/X A11 implant, D 3,5/ L11; Astra Tech EV implant, D 3.6/ L 11, and Xive S plus implant; D 3.8/ L 11, all Dentsply Sirona Implants, Mölndal, Sweden) were subjected to microbiological (*n* = 60) and microscopic investigation (*n* = 15). The examined specimens had distinct system-inherent morse taper (Ankylos C/X and Astra Tech EV) or internal hex (Xive S plus) implant-abutment junctions (IAJ) (Fig. 3).

### Analysis of bacterial translocation

To examine bacterial migration into and out of the restorative conometric coupling system, separate microbiological tests were conducted. First, ten conometric abutments (Acuris, A0, GH 1 to 1.5 mm, Dentsply Sirona implants, Mölndal, Sweden) of each system were connected to the corresponding screw implants (subtotal *n* = 30). This involved the unpacking of the sterile implants and connecting the Acuris abutments to the implants using a new titanium abutment screw and tightening it to the manufacturer’s recommended insertion torque using a pre-calibrated manual torque wrench for each system. Titanium nitride-coated (TiN) stock copings (Acuris Cap, Dentsply Sirona implants, Mölndal, Sweden) were attached manually to the anti-rotation portion of the abutments. The friction fit was obtained by exerting an axially directed load using a dedicated fixation tool with a calibrated striker (Conometric fixation tool; Dentsply Sirona Implants, Mölndal, Sweden). Similar to a clinical setting, final fixation of the TiN copings was verified visually and by manual, non-calibrated pull-off tests. All specimens were finally autoclaved (Autoclave Systec V-40, Systec GmBH, Linden, Germany) and transferred to a Whitley A35 workstation (Whitley A35 Workstation Don Whitley Scientific, Bingley, UK) under anaerobic conditions at 37 °C. For screening of bidirectional bacterial translocation, a mixed bacterial culture suspension consisting of anaerobic early colonizing *Streptococcus mutans* (DSM 20,523, German Collection of Microorganisms and Cell Cultures GmbH, Leibnitz, Germany), moderate colonizing *Actinomyces naeslundii* (DSM 17,233, German Collection of Microorganisms and Cell Cultures GmbH, Leibnitz, Germany), *Fusobacterium nucleatum* (DSM 15,643, German Collection of Microorganisms and Cell Cultures GmbH, Leibnitz, Germany), and late colonizing *Porphyromonas gingivalis* (DSM 20,709, German Collection of Microorganisms and Cell Cultures GmbH, Leibnitz, Germany) species was prepared. The bacteria varied by size with a size ranging from 0.5 to 1–2 µm [11]. The optical density (OD) of the mixed culture was 0.1.

To assess bacterial outgrowth, the occlusal openings of ten Acuris abutments in each of the three different systems were filled with 4 µl of a mixed bacterial culture of anaerobes. The matching TiN caps were seated on the abutments and fixed as previously described. All assembled specimens were then disinfected with 70% aqueous ethanol (EtOH) and transferred to sterile 1.5 ml Eppendorf tubes containing 1 ml bacterial culture medium (CDC) to provide an optimal environment for bacterial colonization. While 4 µl of mixed bacterial culture was filled directly into an Eppendorf tube as a positive control, 4 µl of pure culture medium (CDC) in one of the Acuris abutments served as a negative control. Incubation was maintained at 37 °C for 48, 96, 144, and 192 h. At each specified time interval, a sample of 50 µl was taken from each Eppendorf tube for the analysis of total bacterial count. Each sample underwent DNA preparation (innuPREP DNA Isolation Kit, Analytik Jena AG, Jena, Germany). The respective DNA was quantified by qrt-PCR (quantitative real-time polymerase chain reaction, CFX96 Touch Real-Time PCR Detection System, Bio-Rad Laboratories, Berkeley, California, USA) employing a universal eubacterial 16S rRNA primer (HDA1 GACTCCTACGGAGGCAGCAGT, E1115R AGGGTTGCGCTCGTTGCGG). Universal primer results were specified with appropriate primers for each bacterial strain as listed in Table 1 [10, 12–14].

**Table 1:** Specific primer sequences for qrt-PCR and references of their applicability [10]

Organism	Primer	Primer sequence	Reference of primer applicability
*Porphyromonas gingivalis* *Streptoccocus mutans* *Actinomyces* species *Fusobacterium nucleatum*	CA-PG-F/R MKD-FV/RV ACT-174-F ACT-281-R CA-FN-F/R	AGGCAGCTTGCCATACTGCG ACTGTTAGCAACTACCGATGT GGCACCACAACATTGGGAAGCTCAG GGAATGGCCGCTAAGTCAACAGG GGTCTCTGGGCCGTTACTGA GRCCCCCCACACCTAGTG AGAGTTTGATCCTGGCTCAG GTCATCGTGCACACAGAATTGCTG	Carrouel F. et al., 2016 [12] Hoshino T. et al., 2004 [13] Bizhang M. et al., 2011 [14] Carrouel F. et al., 2016 [12]

To cross-check the findings concerning bacterial translocation out of the conometric components, samples were also tested for bacterial leakage into the conometric system. An additional ten Acuris abutments (subtotal *n* = 30) of the respective systems (Ankylos C/X, Astra Tech EV, Xive S plus) were occlusally filled with 4 µl of culture medium to ensure an optimal environment for bacterial colonization and connected to the Acuris TiN copings. The specimens were transferred to a reaction tube containing 30 ml bacterial mixed culture solution. As a positive control, 4 µl of bacterial mixed culture was filled directly into an Eppendorf tube. Four µl of culture medium (CDC) served as a negative control and replaced the bacterial mixed culture. Over a period of 7 days, a sample of 20 ml of mixed culture solution was taken from the original reaction tube at 48, 96, 144, and 192 h, respectively, and replaced with fresh bacterial culture medium. Simultaneously, at each point of time, two implants were removed from the reaction tube, washed with phosphate buffered saline (PBS), and disinfected with 70% aqueous ethanol (EtOH), followed by removal of the TiN caps from the abutments. The contained solution was processed with a deoxyribonucleic acid (DNA) Isolation Kit (innuPREP DNA Isolation Kit, Analytik Jena AG, Jena, Germany). In concordance to outgrowth testing, the DNA was quantified with qrt-PCR using universal and specific primers for the examined bacterial strains [10, 12–14] (Table 1).

### SEM analysis of conometric connection and luting interface of coping and crown

#### Specimen fabrication

In addition to bacterial leak testing, a total of 15 Acuris specimens for single crown restorations of the three different systems were subjected to scanning electron microscopy, five per system (Ankylos C/X, Astra Tech EV, Xive S plus). Despite different IAJ, the restorative abutment configuration and prosthetic diameter (D 4.5 mm) were identical for all abutments. Thus, the same Acuris TiN copings could be used for all three implant systems. The master cast of a clinical case where the right mandibular first molar had been replaced by a single implant restoration served as origin of the virtual crown design (DentalCAD, Exocad GmbH, Darmstadt, Germany). A temporary implant-supported single crown had been used to precondition the emergence profile of the peri-implant mucosa. Due to the same restorative abutment configuration of all investigated systems, 15 identical monolithic CAD/CAM zirconia crowns were fabricated (Katana, Super Translucent Multi Layered, Kuraray Noritake Dental, Tokyo, Japan). A list of materials and manufacturers is shown in Table 2. Strict adherence to the manufacturer’s recommendations was ensured for the bonding process of the all-ceramic crowns. The inner bonding surface of each crown was conditioned with a ceramic primer (Clearfil Ceramic Primer Plus, Kuraray Noritake Dental, Tokyo, Japan) for 5 s prior to bonding the crowns to the TiN copings with a Bis-GMA/TEGDMA-based cement (Panavia V5, Kuraray Noritake Dental, Tokyo, Japan). The excess of the resin composite cement was removed after the setting process was initiated by a 3-s light polymerization. To prevent an oxygen inhibition layer, the margins were covered with inhibitor gel (Panavia F 2.0 Oxyguard II, Kuraray Noritake Dental) before the curing process was completed by 15 s of light polymerization. Finally, the adhesive joint of each crown-cap unit was carefully polished with silicone polishers. After fabrication of the extraorally cemented crown-coping complexes, the Acuris abutments were connected to the implants as previously described and screwed in place with a dedicated torque wrench. The crown-coping units were then mounted on the anti-rotational part of the abutments and friction-fixed with the calibrated striker tool.

**Table 2:** List of materials, compositions, manufacturers, respective reference no. and quantity used

Material	Composition	Manufacturer	Ref. No	Quantity
Ankylos C/X A11 Implant (D 3,5/ L11)	Titanium grade 2	Dentsply Sirona	3101 0410	5
Astra Tech EV Implant (D 3.6/ L 11)	Titanium grade 2	Dentsply Sirona	25,224	5
Xive S plus Implant (D 3.8/ L 11)	Titanium grade 2	Dentsply Sirona	26 2442	5
Ankylos Conometric Abutment C/ 1.5/0°/Ø4.5/I	Titanium grade 2	Dentsply Sirona	3102 3450	5
Astra Tech EV Conometric Abutment EV/ Ø3.6/1.0/0°/Ø4.5/I	Titanium grade 2	Dentsply Sirona	26,121	5
Xive S plus Conometric Abutment/ Ø3.8/1.0/0°/Ø4.5/I	Titanium grade 2	Dentsply Sirona	32,264,101	5
Ankylos C/X, Astra Tech EV, XiVE S plus Conometric Final Cap, Ø4.5	Titanium Nitride	Dentsply Sirona	31,072,303	15
Ankylos C/X, Astra Tech EV, XiVE S plus Conometric Lab Analogs Ø4.5	Surgical stainless steal	Dentsply Sirona	3107 2020	15
Ankylos C/X, Astra Tech EV, XiVE S plus Conometric Lab Cap Ø4.5	Ti6AL4V-ELI	Dentsply Sirona	3107 2123	15
Fixation Tool Acuris	Surgical stainless steal	Dentsply Sirona	31072,911	1
Katana CAD/CAM Zirconia Crown	ZrO2 + Y2O3: > 98,0 (wt%); pigments < = 2,0 (wt%) Super Translucent Multi Layered (STML)	Kuraray Noritake Dental	A3 125-3182EU	15
Panavia V5	Monomer matrix: hydrophobic aromatic dimethacrylate, hydrophilic aliphatic dimethacrylate, Bis-GMA, TEGDMA; inorganic fillers: silanated barium glass, silanated fluoroaluminosilicate glass, colloidal silica, silanated aluminum oxide (particle size between 0.01 μm and 12 μm, total volume content of inorganic fillers approximately 38 vol%); initiators; accelerators; camphorquinone; pigments	Kuraray Noritake Dental	350008/ 680,008	as manufact recomm
Monobond Plus	Ethanol, silane, methacrylate phosphoric ester	Ivoclar Vivadent	X28859	as needed
Liquid Strip	Glycerin gel	Ivoclar Vivadent	X09458	as needed
Clearfil Ceramic Primer Plus	Ethanol, 3-methacryloxypropyl trimethoxy silane, 10—methacryloyloxydecyl dihydrogen phosphate	Kuraray Noritake Dental	580035	as needed
Panavia F 2.0 Oxyguard II	Glycerin, polyethylene glycol, katalysators, initiators, pigments	Kuraray Noritake Dental	4R0003/ 6J0064	as needed

#### SEM assessment

All samples were processed for scanning electron microscopy (SEM) analysis of polished micrographs. The specimens were embedded in a polyurethane-based model resin (Sherapolan 2:1, Shera Werkstofftechnologie) using UNICLIP specimen holders (Wirtz/Buehler) in a standardized process. Horizontal alignment and cutting to the required specimen sizes were performed automatically with an Accutom-50 precision grinding and cutting machine (Struers). After adjustment to the required parameters (accuracy, ± 5 μm, cut-off wheel width, 0.6 mm), polished thin sections were prepared under water cooling and continuous examination of macro- and microscopic integrity (10 × magnification, photomacroscope, Wild). Subsequent to final inspection, samples were sputtered with Au–Pd for SEM evaluation. Microgaps along the conometric connection and between the luting interface of the TiN coping and all-ceramic crown were measured for the 15 specimens by means of SEM (LEO 1430, Zeiss). In total, 150 SEM measurements, including 90 readings of the conical coupling and 60 recordings of the micro-cement-gap of the restoration, were taken. Distance measurements were evaluated by the same examiner (E.S.) and were made once for each predefined distance. Conical and marginal discrepancies were evaluated at 200 × and 1000 × magnification.

#### SEM readings of conometric connection

**Fig. 4:**
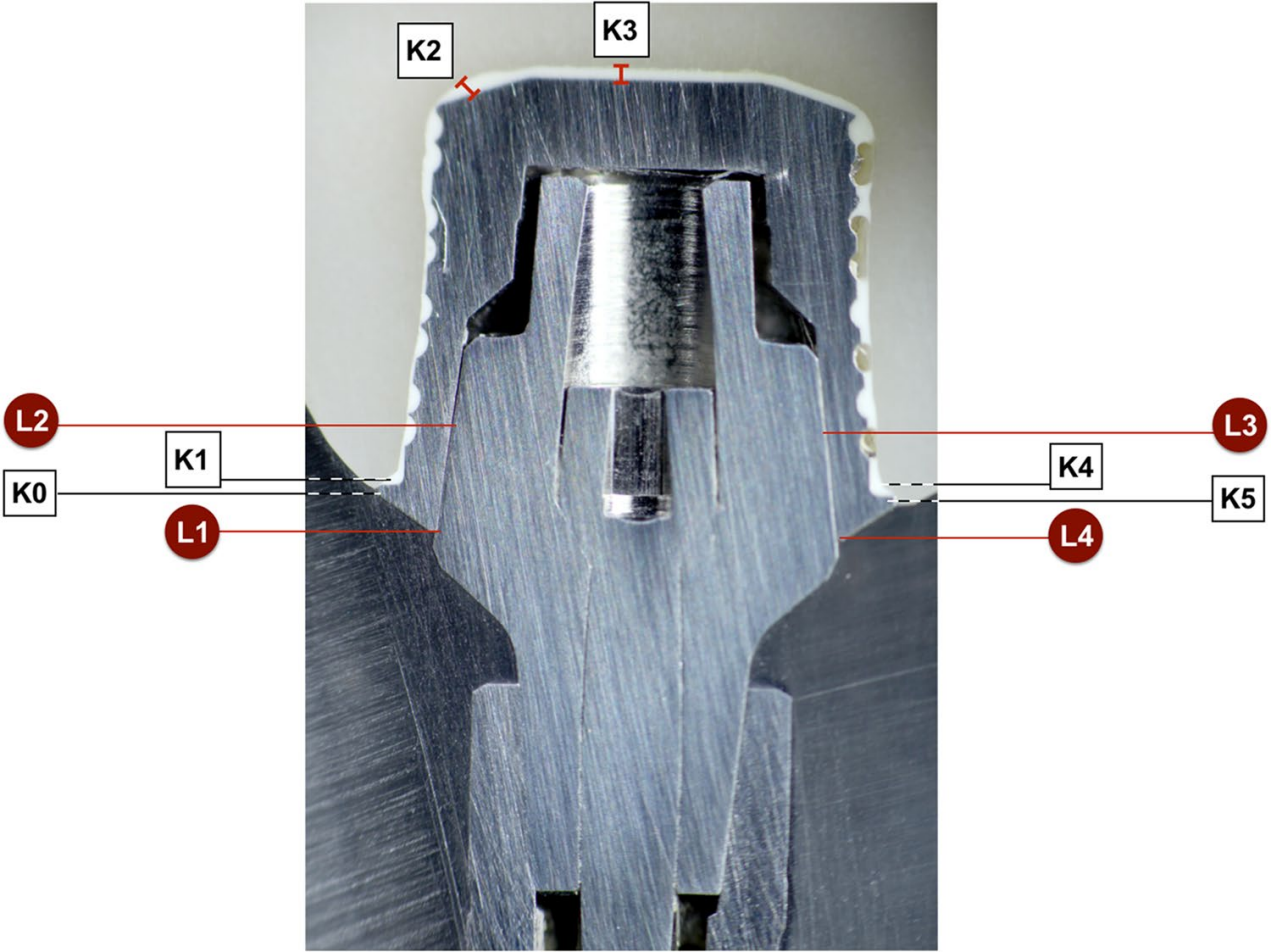
SEM of conometric connection (example: Ankylos C/X specimen) with landmarks L1 to L4. Luting gap and marginal integrity of ceramic crown on TiN coping are displayed at 6 defined reference sites K0 to K5. Points K0 and K5 represent the discrepancy between crown margin and coping after cementation. Landmarks K1 to K4 represent the vertical and horizontal luting gaps inside the crown

Potential microgaps between the TiN coping and titanium Acuris abutment were determined at four prespecified landmarks (L1 to L4) according to distinctive construction characteristics of the conometric connection (Fig. 4). A gap has been defined as the perpendicular distance from the surface of the axial wall of the abutment to the internal surface of the TiN coping. In the clinical situation, landmarks L1 and L4 are located directly within the peri-implant sulcus with potential contact to the surrounding tissues of the oral cavity and were thus grouped as “external gaps.” The remaining landmarks L2 and L3 comprised the mid vertical taper of the Acuris abutment and were consequently recorded as “internal gaps” for SEM analysis. Whereas the external microgaps determine the long-term performance in terms of bacterial leakage entrance, the internal gaps represent the extension of the morse taper junction and are additionally responsible for the mechanical and dimensional properties of the conometric coupling.

#### SEM readings of crown-coping unit

The size of the luting gap and the marginal integrity of the CAD/CAM ceramic crowns on the extraorally cemented Acuris TiN copings were evaluated in the same way at 6 defined reference points (K0 to K5) according to the respective design properties of the prefabricated copings (Fig. 4). While the landmarks K0 and K5 determined the discrepancy of the crown margin and the coping after cementation, the landmarks K1 to K4 represented the vertical and horizontal luting gaps inside the crown.

#### Statistical analysis

Statistical analysis was conducted using SAS 7.4 (SAS Institute Inc., Cary, North Carolina, USA) and BiAS 11.10 (Epsilon Publishing, Frankfurt, Germany). Mean bacterial counts from the qrt-PCR measurement were compared with an exponential-linear model that included implant type and experimental time as fixed effects. The graphical representation is based on the marginal means estimated from the statistical model. Since the data of the SEM measurements were not normally distributed, Wilcoxon-Mann–Whitney tests were performed for pairwise comparison of restorations. Kruskal–Wallis (H) and Chi-square tests (Chi^2^) were used for the comparison of two or more independent groups. The level of significance was set at 5% (*p* < 0.05) for all applied statistical tests.

## Results

### Bacterial outgrowth

**Fig. 5:**
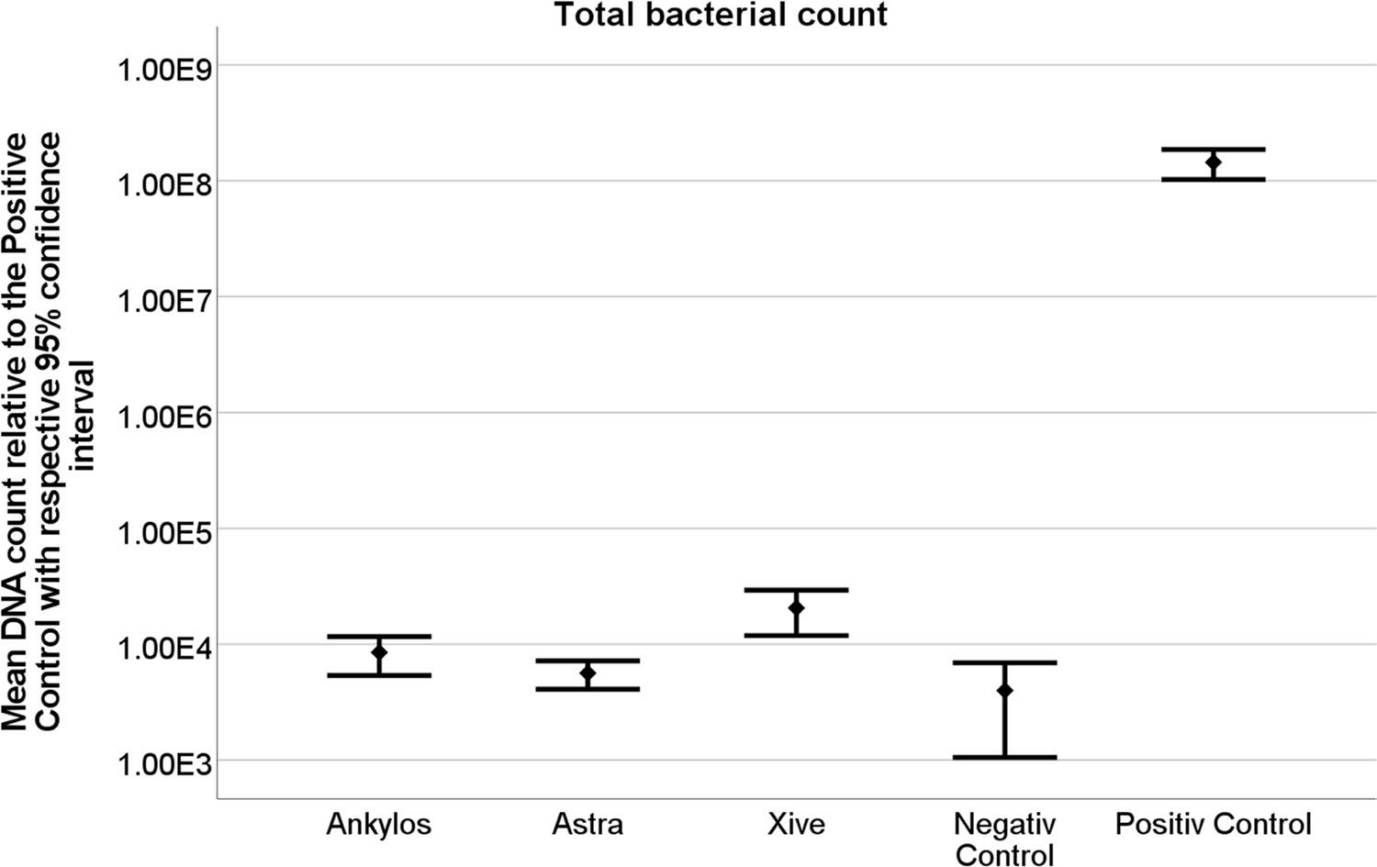
Graphical illustration of statistical results for total bacterial exit. A significant difference of qrt-PCR results between positive control and all three test groups could be demonstrated (*p* < 0.0001). No difference between negative control and all three test groups could be shown

The qrt-PCR results for all Acuris test samples revealed values approaching the negative control for bacterial leakage out of the conometric system. Statistical analysis demonstrated a significant difference for qrt-PCR readings of positive control and all test specimens (*p* < 0.0001) (Table 3, Fig. 5), whereas no difference was found between negative control and test specimens. Comparison of the different test days yielded a significant difference (*p* < 0.0001), although not of clinical relevance (Table 4).

**Table 3:** Comparison of control and test groups demonstrated a significant difference of qrt-PCR results between positive control and test groups (*p* < 0.001), whereas no difference was found between negative control and test groups and between the three different dentals implant systems

Differences of Type / Least Squares Means									
**Group**	**Group**	**Estimation**	**Standard Error**	**DF**	**t-Wert**	**Pr >|t|**	**Alpha**	**Lower**	**Upper**
Anklyos C/X	Astra Tech EV	0.1207	0.1080	140	1.12	0.2653	0.05	− 0.09269	0.3342
Anklyos C/X	Negative Control	0.07961	0.1986	140	0.40	0.6891	0.05	− 0.3130	0.4722
Anklyos C/X	Xive S plus	− 0.1330	0.1080	140	− 1.23	0.2200	0.05	− 0.3464	0.08043
Anklyos C/X	Positive Control	− 4.3264	0.1347	140	− 32.13	< .0001	0.05	− 4.5927	− 4.0602
Astra Tech EV	Negative Control	− 0.04114	0.1986	140	− 0.21	0.8361	0.05	− 0.4337	0.3514
Astra Tech EV	Xive S plus	− 0.2538	0.1080	140	− 2.35	0.0201	0.05	− 0.4672	− 0.04032
Astra Tech EV	Positive Control	− 4.4472	0.1347	140	− 33.02	< .0001	0.05	− 4.7134	− 4.1809
Negative Control	Xive S plus	− 0.2126	0.1986	140	− 1.07	0.2861	0.05	− 0.6052	0.1799
Negative Control	Positive Control	− 4.4060	0.2137	140	− 20.61	< .0001	0.05	− 4.8286	− 3.9834
Xive S plus	Positive Control	− 4.1934	0.1347	140	− 31.14	< .0001	0.05	− 4.4597	− 3.9272

**Table 4:** Comparison of test and control group had a significant effect on the results of bacterial growth (*p* < 0.001). A significant difference for mean bacterial count on different test days was observed

Type III test of effects				
Effect	No. DF	Den DF	F-value	Pr > F
Type	4	140	332.65	< .0001
Day	1	140	40.72	< .0001

### Bacterial ingrowth

**Fig. 6:**
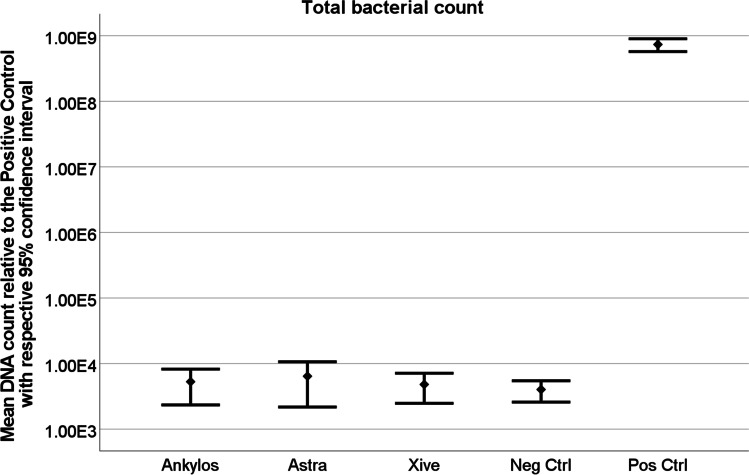
Graphical illustration of the statistical results for total bacterial entry. While a significant difference in qrt-PCR results was shown between the positive control and all three test groups (*p* < 0.0001), no difference could be detected between the negative control and the test groups

Also, the qrt-PCR results for potential bacterial entry into the conometric system remained negative for all specific primers tested on all three implant systems and were significantly different from the positive control (*p* < 0.0001) (Fig. 6).

### SEM readings of microgap dimensions of conometric connection

**Fig. 7:**
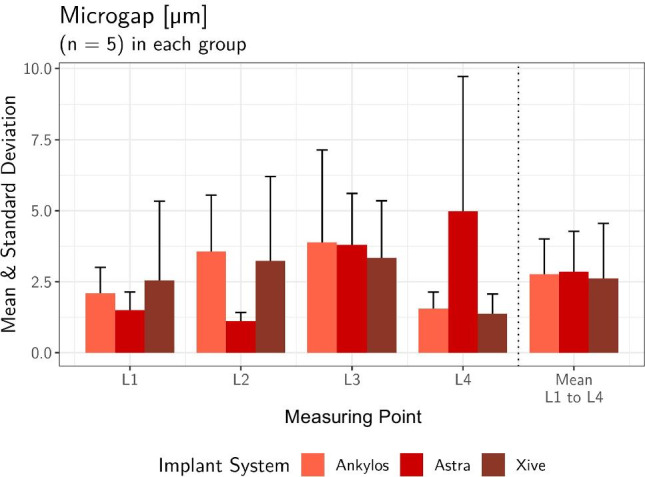
Bar graph of the recorded mean conometric microgap dimensions at landmarks L1-L4 for each individual system and total value

**Fig. 8:**
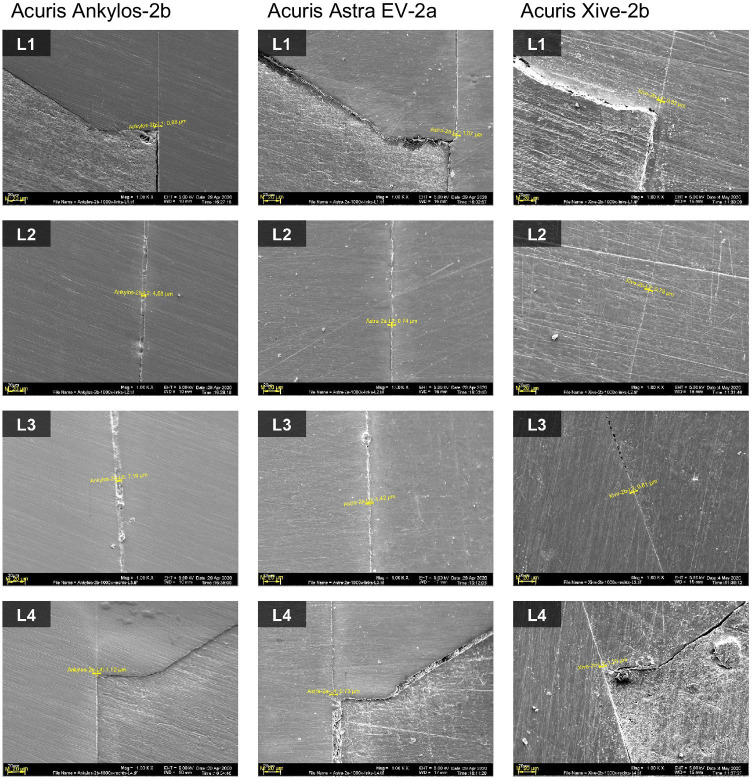
Exemplary SEM images of the three systems examined at 1000 × magnification, showing the punctuate microgaps of the conometric connection at landmarks L1 to L4. Reference points L1 and L4 refer to the apically located areas of the coping margin (external gaps). Landmarks L2 and L3 represent the mid-vertical taper of the Acuris abutment (internal gaps)

Despite the planar contact along the cone-in-cone interface, miniscule punctate microgaps could be recorded in SEM analysis at the predefined reference sites L1 to L4 of the conometric connection. The mean external microgap for all abutment specimens averaged 2.04 ± 1.67 µm (min. 0.83 µm/max. 7.43 µm) at the landmarks L1 and 2.64 ± 3.1 µm (min. 0.72 µm/max. 11.8 µm) at the contralateral reference sites L4 (Table 5). The internal mid-vertical microgaps L2 and L3 reached a mean value of 2.64 ± 2.22 µm (min. 0.74 µm/max. 7.67 µm) and 3.67 ± 2.28 µm (min. 0.81 µm/max. 7.67), respectively. When comparing the three systems, there was no significant difference in the microgap size of the respective landmark investigated (Kruskal–Wallis *p* > 0.05). Table 6 and Fig. 7 list the mean microgap dimensions of all conometric connections at four reference sites for each system individually and collectively. Figure 8 shows exemplary SEM images at landmarks L1 to L4 of the three systems examined at 1000 × magnification.

**Table 5:** Overall mean values of gap dimensions at the conometric reference sites (L1–L4) for all specimens tested (total *n* = 15), standard deviation, median, minimum, and maximum

Location	Mean	SD	Median	Min	Max
Microgap L1	2.04	1.67	1.52	0.826	7.43
Microgap L2	2.64	2.22	1.98	0.744	7.67
Microgap L3	3.67	2.28	3.35	0.805	7.67
Microgap L4	2.64	3.1	1.48	0.716	11.8
Mean all L	2.75	1.44	2.24	0.918	5.84

**Table 6:** Mean microgap dimensions, standard deviation, and statistical significance of all conometric connections at four reference sites (L1–L4) for each system individually and collectively

	Ankylos C/X (*n* = 5)	Astra Tech EV (*n* = 5)	Xive S plus (*n* = 5)	Test
Location	Mean ± SD	Mean ± SD	Mean ± SD	*p* value
Microgap L1	2.09 ± 0.92	1.50 ± 0.638	2.54 ± 2.79	0.619
Microgap L2	3.56 ± 1.99	1.12 ± 0.294	3.24 ± 2.96	0.065
Microgap L3	3.89 ± 3.25	3.80 ± 1.81	3.33 ± 2.02	0.932
Microgap L4	1.55 ± 0.58	4.99 ± 4.74	1.38 ± 0,69	0.310
Mean all L	2.77 ± 1.23	2.85 ± 1.42	2.62 ± 1.93	0.827

### SEM readings of cement gap dimensions of crown-coping complex

**Fig. 9:**
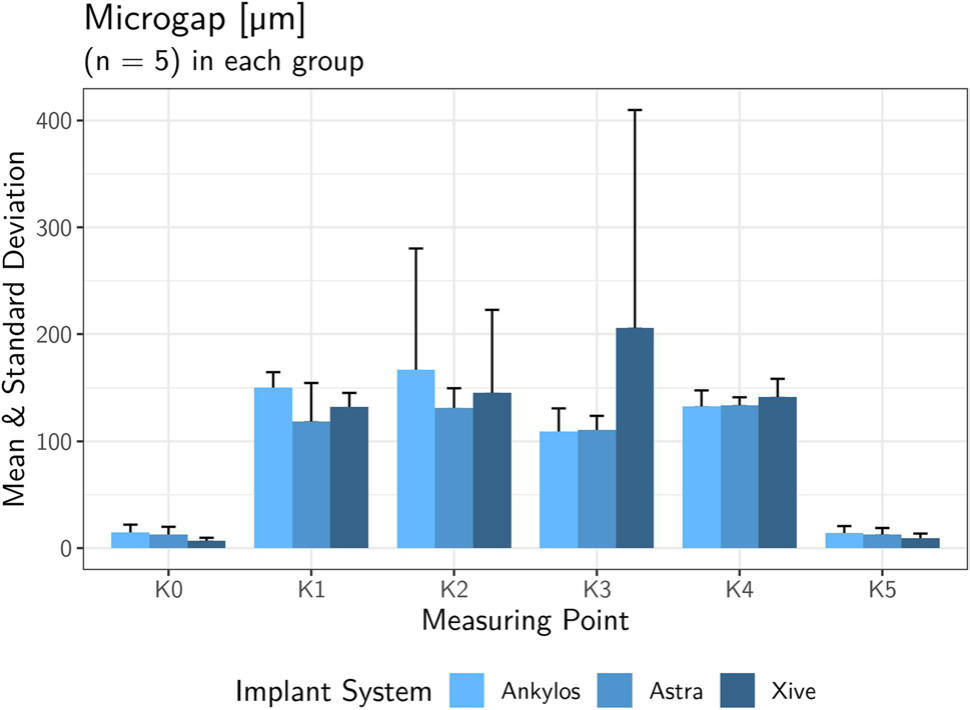
Bar graph of the mean external (K0/K5) and internal crown-coping cement gaps (K1–K4) of the three groups of implant-abutment systems

**Fig. 10:**
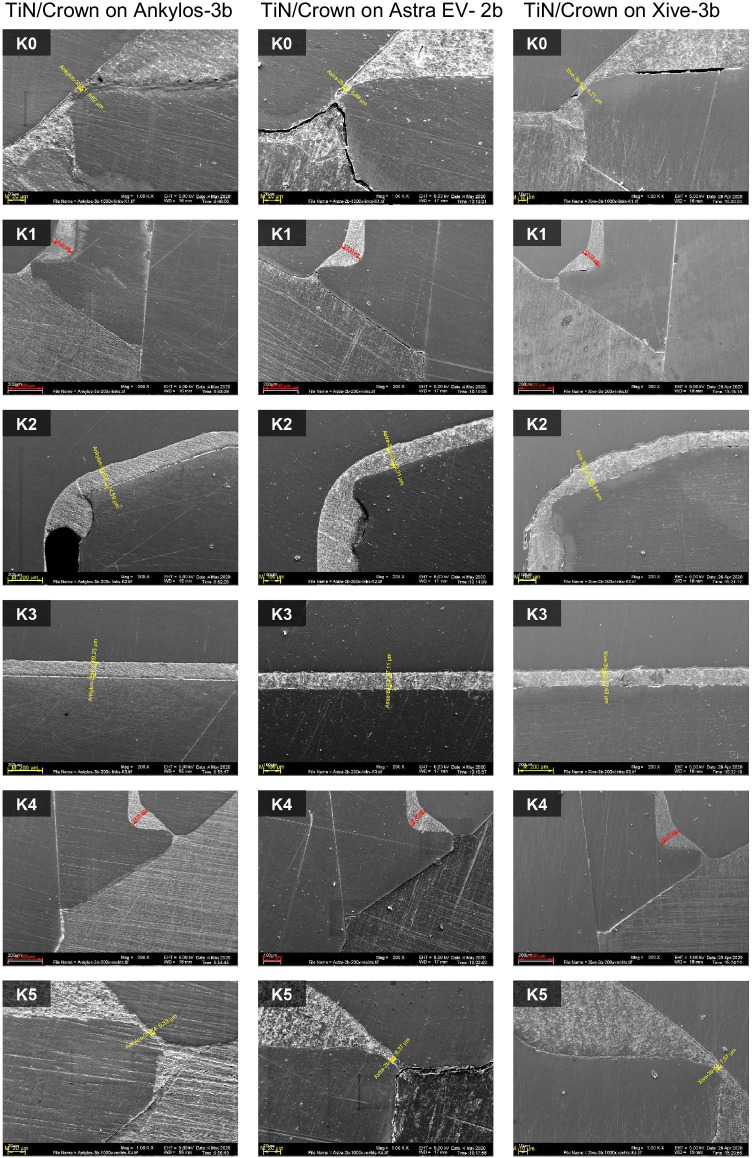
Exemplary SEM images showing the measurements for cement gap and marginal integrity of the ceramic crowns on cemented Acuris copings at reference points K0 to K5. Landmarks K0 and K5 determine the marginal discrepancy of the crown and the coping after cementation at 1000 × magnification. Landmarks K1 to K4 represent the vertical and horizontal luting gaps inside the crown at 200 × magnification

The mean marginal opening of the all-ceramic crowns at the reference points K0 and K5 measured 11.7 ± 5.93 µm (min. 5.25 µm/max. 22.8 µm) for all samples, while the internal cement gap widths amounted to 135 ± 14.6 µm (min. 96.8 µm/max. 156 µm) for landmarks K1 and K4 and 145 ± 84.5 µm (min. 83.3 µm/max. 423 µm) for K2 and K3, respectively (Table 7). Despite the evident differences between the mean external (K0 and K5) and internal microgaps (K1 to K4) (Chi^2^ = 24.1; *p* < 0.001), none of the implant systems showed systematically higher or lower values than the other groups (Fig. 9). The measured cement gap dimensions of all 15 specimens at six reference points for each individual system are shown in Table 8. A comparison among the respective crown coping landmarks K0 to K5 of the three implant abutment systems showed no statistically significant difference with respect to the mean cement gap (Kruskal–Wallis *p* > 0.05). Figure 10 shows exemplary SEM images of cement gap measurements and marginal integrity of the ceramic crowns on the cemented Acuris copings at 200 × magnification.

**Table 7:** Overall mean values of cement gap sizes (K0-K5) for all specimens tested (total *n* = 15), standard deviation, median, minimum, and maximum

Location	Mean	SD	Median	Min	Max
Microgap K0	11.4	6.66	11	3.91	24.1
Microgap K1	134	25.6	137	56.5	167
Microgap K2	148	75.4	127	37.8	293
Microgap K3	142	119	118	84	570
Microgap K4	136	13.3	134	114	161
Microgap K5	12.1	5.69	10.3	5.3	21.6
Mean K0 & K5	11.7	5.93	10.7	5.25	22.8
Mean K1 & K4	135	14.6	135	96.8	156
Mean K2 & K3	145	84.5	122	83.3	423
Mean all K	97.2	29.7	87.5	76.1	191

**Table 8:** Comparison of the three implant-abutment systems in terms of mean cement gap widths, standard deviation and statistical significance at all six measuring landmarks (K0 to K5) for each system tested

	Ankylos C/X (*n* = 5)	Astra Tech EV (*n* = 5)	Xive S plus (*n* = 5)	Test
Location	Mean ± SD	Mean ± SD	Mean ± SD	*p* value
Gap K0	14.7 ± 7.33	12.5 ± 7.45	6.99 ± 2.68	0.174
Gap K1	150 ± 14.6	119 ± 35.9	132 ± 13.2	0.141
Gap K2	167 ± 113	131 ± 18.4	146 ± 77.1	0.961
Gap K3	109 ± 21.8	111 ± 13.4	206 ± 204	0.619
Gap K4	133 ± 14.9	134 ± 7.41	142 ± 16.9	0.651
Gap K5	14.2 ± 6.48	12.8 ± 6.01	9.24 ± 4.38	0.392

## Discussion

In an effort to minimize inflammatory responses and thereby maximize bone stability around the implant platform, numerous in vivo and in vitro studies have demonstrated the influence of the implant-abutment (I-A) microgap on marginal leakage [15–24]. This is in contrast to the limited data available on the fit and potential for microbial leakage at conometric prosthetic connections [7, 10], Clinical evidence of bacterial sealing of the taper coupling at the restorative level is lacking to date. Marginal and internal fit at the abutment-prosthesis interface are critical determinants as they are directly related to bio-integrity, microbial sealing, and maintenance of peri-implant tissue health [25]. As a consequence, the current in vitro study investigated the presence of microgap formation and bacterial translocation at the cone-in-cone interface of three different implant abutment systems for single crown restoration. In addition, the internal fit and marginal integrity of all-ceramic crowns on the matching tapered copings were determined.

The qrt-PCR results of the microbiological tests indicate that the Acuris conometric interface of all three implant systems investigated does not allow for bacterial translocation under unloaded conditions. None of the systems studied (Ankylos C/X, Astra Tech EV, Xive S plus) exhibited any significant bacterial leakage into or out of the conometric junction. Thus, the hypothesis that the Acuris coupling precludes bacterial translocation irrespective of the implant system type can be regarded as accepted. In terms of methodology, the application of qrt-PCR has been previously proven to be an accurate screening tool with a high diagnostic sensitivity for the determination of microbial migration in a pilot study by the authors [10]. Providing consistent positive and negative controls through both directions of the assay setup rendered reliable results. The testing period for bidirectional bacterial translocation was 7 days. Longer observation periods are discouraged due to an increase in false negative findings [26]. The four most common representatives of the oral microbiome (*Streptococcus mutans, Actinomyces naeslundii, Fusobacterium nucleatum, Porphyromonas gingivalis*) were included in the tested bacterial mixed cultures. These bacteria are facultative pathogens and are associated with caries, mucositis, periodontitis, and peri-implantitis [27]. Setting parameters of the cultures were guided by the German Collection of Microorganisms and Cell Cultures (Leibniz Institute DSMZ, Braunschweig, Germany). The culture medium was renewed every 48 h to ensure optimal conditions for bacterial growth as described in previous studies [12]. Adequate bacterial growth environment was confirmed by positive qrt-PCR results for each positive control at all time points. Given the results of the bacterial assays, the principal acceptance that sealing and retention of morse taper connections are achieved by wedge action [17, 23] may also be applied to the sealing efficiency of conometrically seated prosthetic components at the abutment-coping interface. In this context, it is important to note that the friction-based tapered coupling requires a fully seated matrix on the abutment. Incorrectly mounted conometric components will cause poor sealing and may present a risk for bacterial leakage. Within a clinical setting, incomplete retention of the crown-coping-unit would induce occlusal disturbances, a friction deficit, and instantaneous dislocation of the crown. Causes for clinically inferior crown fit and insufficient retention may include tight proximal contacts or a pronounced emergence profile design of the soft tissues.

A secondary objective of the study was to optically determine the fit of the conometric coupling as well as the internal fit and marginal integrity of cemented CAD/CAM crowns on the matching Acuris TiN copings by means of SEM. Despite the fact that the comprehensive microbiological examination in a double verification setup failed to demonstrate microbial leakage from or into the Acuris abutment system, SEM analysis was able to detect minute punctate microgaps at predefined reference sites of the conometric connection. The mean outer microgap for all abutment specimens clinically positioned just within the peri-implant sulcus was 2.04 and 2.64 µm, respectively. The inner mid-vertical microgaps reached a mean value of 2.64 and 3.67 µm, depending on the measuring point. When comparing the respective measuring points, no significant difference in the microgap dimensions between the systems could be detected. The first part of the null hypothesis, which stated that the conometric interface exhibits no detectable microgap microscopically, could thus be considered rejected. In contrast to the minimal punctual gaps of the conometric joint, considerably larger cement gaps were observed at the restorative interface between the crown and the Acuris TiN coping. Whereas the marginal opening of the CAD/CAM ceramic crowns averaged 12 µm for all specimens, the mean value for the internal cement gap was as high as 145 µm. The present results confirm the findings of 3D evaluations demonstrating enlarged internal spaces at the angles of milled restorations. This phenomenon may be related to constraints in milling precision caused by the size of the milling burs [28, 29]. Despite the obvious differences between marginal deficiencies and inner microgaps, none of the tested implant systems exhibited a significant difference with respect to the mean cement gap. Thus, the second part of the null hypothesis, which postulated no difference in internal fit and marginal integrity between the tested systems, could not be rejected.

While the importance of internal crown fit and, in particular, its marginal integrity is generally agreed upon in terms of clinical survival and restoration quality, views on the clinical relevance of the magnitude of marginal discrepancies are controversial. The marginal fit of conventionally fabricated all-ceramic crowns was found to range from 30 to 160 μm [30–32]. Substantial marginal discrepancy in cemented restorations increases the layer thickness of the luting material exposed to oral fluids, which in turn may result in cement dissolution and marginal leakage. The difficulty of removing excess cement when the marginal gap exceeds 100 μm has been pointed out in some studies [33]. Wolfart et al. reported a significant increase in the median marginal deviation of pressed lithium disilicate crowns from 96 to 130 μm due to cementation [34]. Inadequate marginal adaptation increases plaque accumulation and alters the distribution of microbiota, leading to inflammation of periodontal tissues around teeth and peri-implant infections around implants [25, 35, 36]. Bone loss and ultimate breakdown of osseointegration may occur and be responsible for clinical failure of fixed implant restorations [37]. The precision of fit of a restoration also affects the long-term stability of all-ceramic crowns [38, 39]. A causal relationship between increased cement thickness and reduced bending strength of ceramics has been documented [40, 41]. Restorations manufactured by computer-aided design/computer-assisted manufacturing (CAD/CAM) techniques displayed marginal discrepancies less than 100 μm [42, 43] and improved marginal integrity [30, 44]. These findings are in agreement with the results of the current study for internal fit of the crown and its marginal discrepancies. However, for a comparison, the different materials, measurement methods, and restoration types (FPDs vs. SCs) must be taken into account. Despite the fact that a digitally assisted fabrication process enhances the fit of all-ceramic frameworks, microscopic evidence indicates that a gap and correlating misfit between the ceramic crown and its respective abutment cannot be fully avoided [28, 29, 45]. In this regard, it should be noted that the marginal gap widths for all-ceramic restorations increase proportionally to the final curvature line, irrespective of the ceramic material [46].

Overall, the methods used for sample production and evaluation should provide a realistic representation of the clinical situation in the fixation of conometric morse taper connections for the retention of implant-supported single crowns (SCs). A limitation associated with the SEM analysis was that the landmarks selected for gap measurements may not have been truly representative for the overall fit of the components. Since a complete measurement over the entire interface area was neither practical nor reasonable, the data obtained can, however, be considered representative. A further constraint of the present study relates to the conflicting findings regarding the complete seal against micro-leakage, on the one hand, and the evidence of punctiform gap formations at the conical interface of the morse-taper junction on the other hand. The reason for this fact may be due to the particular design of the Acuris connection. Unlike conventional conical connections, where the joint surfaces contact each other in a full planar configuration, the Acuris conometric connection is designed in such a way that it has two counter bearings. An annular mating surface (first counter bearing) is provided at the opening area of the TiN coping. The contact zone between the abutment index and the coping has a cylindrical contact surface that acts as a second counter bearing. The ring-shaped mating surface is designed to prevent bacterial translocation, while the cylindrical pressure of the mounting surface provides resistance to shear forces during masticatory movements. A flat contact surface along the entire taper is deliberately omitted, as the removal forces would consequently be difficult to adjust or to control clinically. The SEM images of the present analysis reflect this engineering principle under the current conditions of use (Fig. 4). The matrix material of the titanium nitride copings comprises grade 4 titanium, while the Acuris abutment itself is made of a grade 5 titanium alloy (Ti 6Al-4 V ELI). Due to the required grinding and preparation processes of the specimens for SEM analysis, manufacturing-related smearing of the softer TiN coping material may have occurred at the interface. Despite adherence to high-quality precautions, it cannot be completely ruled out that possible miniature artifacts were misinterpreted as microgaps during SEM evaluation. To put the relevance of the obtained in vitro results into a clinical perspective, further studies are needed to determine the long-term outcome of peri-implant tissue health of conometrically seated SCs.

## Conclusions

Within the limitations of the present bidirectional in vitro study, no bacterial leakage from or into the Acuris abutment of 3 different implant systems could be detected upon microbiological examination. SEM analysis revealed tiny punctate microgaps at the most apical point of the conometric connection with an average width of 2 to 3 µm for all systems tested. Considerably larger cement gaps were observed at the restorative interface between the all-ceramic crown and the matching Acuris TiN coping. The marginal discrepancies of the CAD/CAM crowns averaged 12 µm across all specimens, while the mean value for the internal cement gap amounted for up to 145 µm.
